# A quantitative evaluation study of China’s Long-Term Care insurance policies based on the PMC index model: A case study of 16 pilot policy texts

**DOI:** 10.1371/journal.pone.0321057

**Published:** 2025-04-11

**Authors:** Yuzi Wang, Weizheng Wang, Yujie Zhang, Yuan Zeng

**Affiliations:** 1 School of Social Sciences, Tsinghua University, Beijing, Beijing, China; 2 School of Philosophy and Social Development, Shandong University, Jinan, Shandong, China; 3 School of Social Development and Public Policy, Fudan University, Shanghai, Shanghai, China; 4 Gongyi Municipal Committee of the Communist Party of China Social Work Department, Zhengzhou, Henan, China; Erasmus University Rotterdam, NETHERLANDS, KINGDOM OF THE

## Abstract

China’s Long-Term Care Insurance (LTCI) system is designed to address the caregiving needs of elderly individuals with disabilities and dementia, focusing on long-term care to ensure adequate survival and quality of life for disabled individuals. This study employs the PMC index model to evaluate LTCI policies in 16 pilot cities, including Changchun and Qingdao, using keyword extraction and social network analysis on the latest policy documents from these cities. The study revealed the following findings: (1) Among the 16 cities, only Qingdao’s policy achieved a “perfect” rating, with two policies rated as “excellent,” 11 as “acceptable,” and two as “poor.” (2) While most cities allow the involvement of commercial insurance in LTCI administration, there is a lack of clear policy direction for the assessment and service provision by commercial insurers. (3) Dementia care receives significantly less attention compared to physical disabilities. (4) Most cities have underdeveloped financing mechanisms, and family caregiving services are undervalued. Moreover, an analysis of representative policies based on the PMC surface indicates substantial differences between the pilot cities, with Qingdao’s “perfect” policy serving as a model for future LTCI development. The study offers several recommendations: (1) Improve caregiver support policies to enhance family caregiving services. (2) Expand funding sources to increase the equity of LTCI financing. (3) Allocate insurance policy resources more effectively to gradually eliminate policy barriers. (4) Increase the focus on dementia care and clarify the criteria for assessing disability. (5) Strengthen the preventive function of LTCI and progressively expand its coverage. This research provides critical insights into the ongoing development of China’s LTCI system and proposes viable strategies for promoting equity and sustainable growth.

## 1. Introduction

According to the results of the fourth sample survey on the living conditions of elderly people in urban and rural China, 18.3% of the elderly population in 2015, approximately 40.63 million people, were either fully or partially disabled. The predicts that from 2020 to 2050, the number of elderly individuals with disabilities will continue to rise due to the ongoing expansion of the elderly population [1]. In response to the increasing burden of caregiving for severely disabled elderly individuals, the gradual establishment of a long-term care insurance (LTCI) system and the improvement of an integrated medical and elderly care service system have become crucial components of China’s proactive strategy to address population aging. The LTCI system is designed to meet the caregiving needs of elderly individuals with disabilities and dementia, focusing on the provision of long-term care to ensure better survival security and quality of life for disabled individuals. The establishment of the LTCI system also helps to address the non-professional nature of elderly care, alleviating the economic burden on families and caregivers. At the same time, it facilitates the downward transfer of high-quality medical resources, optimizes the allocation of caregiving resources, and promotes the development of an intelligent and healthy elderly care industry [2].

In 2016, the Office of the Ministry of Human Resources and Social Security issued the “Guiding Opinions on Conducting Pilot Programs for the Long-Term Care Insurance System” (Document No. 80 [2016] of the Ministry), selecting 15 cities to initiate national-level LTCI pilot programs. In September 2020, the National Healthcare Security Administration, in collaboration with the Ministry of Finance, released the “Guiding Opinions on Expanding the Pilot Programs for the Long-Term Care Insurance System” (Document No. 37 [2020] of the Healthcare Security Administration), adding 14 additional pilot cities. This expansion aims to explore a framework for an LTCI system suited to China’s national context on a broader scale. The pilot cities have actively implemented LTCI policy trial plans, achieving some success in practice. However, challenges such as limited service content, underdeveloped financing mechanisms, and immature management models have emerged. At the same time, there are significant differences in the implementation effectiveness of the policies across the pilot cities. Are these issues related to the design of the LTCI system? What differences exist in the policy schemes among the pilot cities? Are the outcomes of LTCI policy implementation influenced by other factors? Based on these questions, this paper applies the Policy Modeling Consistency (PMC) index model to establish an LTCI policy evaluation index system. Through PMC surface diagrams and a comparative analysis of LTCI development in countries with more advanced systems, this study critically examines the strengths and limitations of LTCI policy design, providing scientific references for the sustainable development of the LTCI system.

## 2. Literature review

### 2.1 The concept of long-term care insurance and international experiences

Long-Term Care Insurance (LTCI) is a system designed to provide long-term care services for individuals who are unable to manage daily living activities due to age, illness, or functional disabilities [3]. The core of LTCI lies in providing financial support for long-term care services through social or commercial insurance mechanisms. As global aging trends intensify, the demand for long-term care is steadily increasing, leading many countries to design and implement various forms of LTCI systems according to their specific national contexts. Summarizing and comparing these international experiences offers valuable insights into the design and effectiveness of LTCI systems.

#### 2.1.1 . The long-term care insurance system in Germany.

Germany was the first country in the world to introduce a long-term care insurance system, officially implemented in 1995 as part of its social security system. A key feature of Germany’s LTCI is its mandatory and intergenerational solidarity principle, requiring all residents covered by statutory health insurance to participate in LTCI, with premiums shared between employers and employees [4]. The system is built on the principle of “intergenerational support,” where the contributions of current workers fund care services for those in need. Germany’s LTCI covers a wide range of services, including home care, institutional care, and community-based care. Notably, the system prioritizes home care to reduce pressure on public care institutions and encourages family members to be actively involved in caregiving. The government provides cash benefits to individuals requiring long-term care, allowing beneficiaries to choose between using the funds for family-based care or purchasing professional care services [5]. Furthermore, the care needs assessment mechanism within Germany’s LTCI system is highly regulated, with care services only provided following evaluation by professional assessment bodies to ensure that recipients are genuinely unable to care for themselves, whether elderly or disabled. Care services are categorized into different levels based on the degree of need, ensuring that those with higher needs receive greater support. Germany’s LTCI system not only alleviates the burden of caregiving in an aging society but also significantly shares the financial burden of care, reducing the economic strain on families [6].

#### 2.1.2 The long-term care insurance system in Japan.

Japan’s Long-Term Care Insurance (LTCI) system was officially implemented in 2000, drawing from Germany’s model but with distinct features in its policy design. Unlike Germany, Japan’s LTCI covers all residents aged 40 and above, rather than being limited to insured individuals. The premiums are jointly funded by residents over 40, local governments, and the central government, ensuring a diversified funding source and the sustainability of the system [7]. A key feature of Japan’s LTCI is its mandatory and universal nature, requiring all residents aged 40 and above to pay LTCI premiums, which gives the system broad coverage. Japan’s long-term care service system emphasizes the integration of community services with home care, striving to keep the elderly out of institutional care facilities. The government provides financial and policy support to promote the development of community-based elderly care services, enhancing the accessibility and quality of home care. Similar to Germany, Japan has established a standardized care needs assessment system, which assigns different levels of services and financial support based on the degree of care needed by the insured. Additionally, the Japanese government places great emphasis on the training and management of care service workers, ensuring service quality through a stringent certification system [8]. In terms of policy design, Japan’s LTCI is government-led, with a strong emphasis on public financial support to ensure that all elderly individuals requiring care receive basic services [9].

#### 2.1.3 The long-term care insurance model in the United States of America.

In contrast to the social insurance models of Germany and Japan, the long-term care system in the United States of America primarily relies on private long-term care insurance and the public Medicaid program. Private long-term care insurance is mainly targeted at high-income individuals, and its market-driven nature has resulted in low enrollment rates, with only a small portion of middle- and upper-income families able to afford the high premiums of private insurance [10]. Furthermore, the coverage provided by private long-term care insurance is often limited, typically covering only a portion of care costs, leaving many elderly individuals’ care needs unmet. In contrast, Medicaid, as a federal public medical assistance program, covers a large portion of long-term care costs for low-income elderly individuals. However, Medicaid’s coverage is limited to individuals who meet specific financial eligibility criteria [11]. The U.S. long-term care system faces challenges such as insufficient funding, inadequate service provision, and a fragmented system. Although the government provides some level of support for long-term care, the lack of a unified national system leads to significant variations in care quality and coverage across states. Compared to the more cohesive systems in Germany and Japan, the U.S. LTCI system is more fragmented and underdeveloped, which contributes to greater financial pressure and resource shortages in the field of long-term care in the United States [12].

A comparison reveals that the long-term care insurance systems of different countries exhibit significant differences in terms of funding sources, coverage, and service models. The social insurance models in Germany and Japan are characterized by universal coverage and broad-based social solidarity [13], whereas the United States of America relies more on market-driven mechanisms [14]. These international experiences offer valuable insights for the design and optimization of LTCI systems globally, including lessons on funding mechanisms, coverage, and service delivery models.

### 2.2 The development and current status of China’s long-term care insurance policy

#### 2.2.1 The construction of the long-term care insurance mechanism.

From the perspective of long-term care insurance (LTCI) policy, research based on the social welfare policy analysis framework has examined China’s LTCI system in major pilot cities through the lens of four key elements: social distribution, types of service provision, delivery systems, and financial models. Findings indicate that issues such as evaluation tools and service supply accuracy have been persistent [15–17]. He [18] conducted a comprehensive study on the financing mechanism through literature review and in-depth interviews, proposing a multi-channel independent financing mechanism. Other innovative studies have also gained attention, such as those exploring the role of social forces as important sources of LTCI funding and service provision [19–20] and new models like the “gradual coverage of insured individuals” and “fixed financial responsibility” exemplified by Jinjiang’s LTCI pilot program [21].

In terms of covered populations, Germany and other countries generally have broader coverage in both the insured and the beneficiaries compared to China [22–24]. For example, in the LTCI trial in Shanghai, researchers found that the citywide six-level elderly needs assessment system had issues with overly detailed classifications [25]. Furthermore, China is actively exploring flexible LTCI mechanisms tailored to its own context. Wang [26] conducted theoretical and empirical studies, suggesting that the design of the LTCI system should take into account the characteristics of demand that influence service needs. In addition, scholars have used descriptive statistics and ordinal logistic regression to analyze satisfaction and influencing factors among LTCI beneficiaries in Shangrao, Jiangxi Province, showing a generally high level of satisfaction [27].

#### 2.2.2 The development of long-term care insurance operations.

In terms of operational models, Germany and Japan’s “independent insurance” models represent a shift from China’s pilot LTCI, which was initially attached to the medical insurance model [28–30]. Additionally, studies have affirmed the unique local practices of integrating medical and elderly care and the “mutual assistance” model of LTCI that features the interplay between family and community care [31,32]. However, operational outcomes have revealed various problems in China’s LTCI system, including financing sources, the identification of beneficiaries, and the supply of care resources and services [33,34]. Yang [35] studied the operational effectiveness of the pilot in Chengdu, employing interviews and data analysis, and found inefficiencies such as wasted resources and underutilization of services on both the supply and demand sides.

#### 2.2.3 Long-term care insurance service models.

Many scholars have applied content analysis methods to study service models in LTCI pilot programs. For example, researchers using deductive content analysis have examined the characteristics of LTCI pilot programs in 14 cities across China and found that all cities covered institutional care, while most (except for Changchun, Chengde, and Ningbo) also included home-based care [36–40]. Subsequently, researchers expanded the scope to include both the first 15 pilot cities and the 13 new pilot cities, comparing service models and categorizing them into institutional care, home care, and non-conventional models tailored to local conditions [41]. In terms of service progress, some scholars have analyzed the current state of professional care personnel, disability assessment, care ratings, and service projects, with the aim of establishing standards for personnel qualifications, work processes, and service quality [42]. Huang [43] conducted a quantitative analysis of care resources in Chengdu, one of the first pilot cities, using Lorenz curves, Gini coefficients, and Theil indices to evaluate equity.

In terms of LTCI policy evaluation, various studies have employed quantitative methods. Zheng [44] developed a multi-tier evaluation framework for LTCI in Qingdao, Chengdu, and Shijingshan District in Beijing, incorporating core policies, management, and supporting linkages into 3 primary indicators, 10 secondary indicators, and 20 tertiary indicators. However, studies utilizing specialized policy tools for comprehensive evaluation of LTCI policies remain limited, with only a few researchers employing the Policy Modeling Consistency (PMC) index model for a thorough exploration of LTCI policy[45–48].

In summary, previous studies have largely focused on qualitative methods such as interviews, though some have utilized quantitative models and text analysis, yet no systematic framework has emerged. Much research has concentrated on mechanism construction, operational development, and service models, but systematic analysis of LTCI policy texts is lacking. Similarly, while some studies have applied the PMC index model to analyze LTCI policies, an academic framework has not yet been fully developed. This study, therefore, utilizes the PMC index model to scientifically evaluate China’s LTCI policies and mechanisms, aiming to provide new insights for the improvement of the LTCI system.

## 3. Construction and analysis of the PMC index model

The Policy Modeling Consistency (PMC) index model is a policy evaluation tool that includes both economic and non-economic variables. It can be used to analyze the internal characteristics of any policy and scientifically quantify the advantages and disadvantages of different policies. In this study, binary values were assigned to all variables, and the PMC index was calculated to evaluate the institutional design of the policies.

### 3.1 Policy selection and text mining

**Fig 1:**
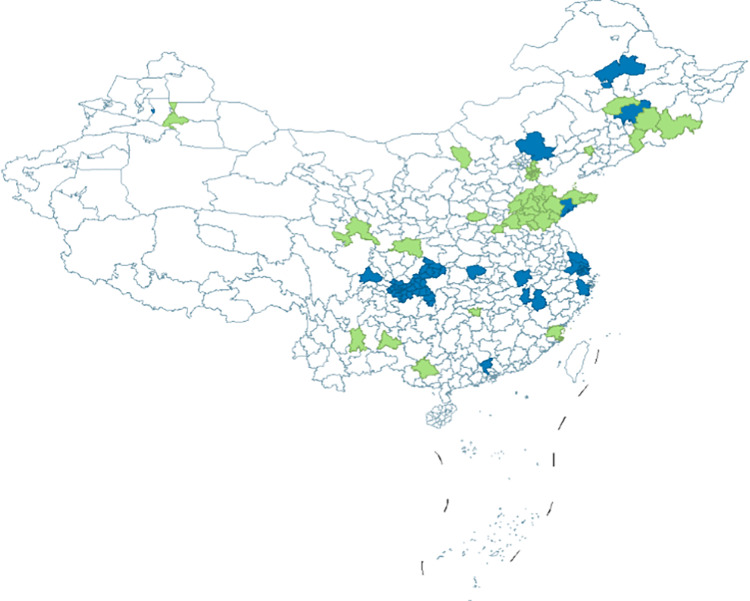
Two batches of pilot cities for the long-term care insurance system.

Note: Blue represents the first batch of pilot cities, and green represents the second batch.

In June 2016, the Office of the Ministry of Human Resources and Social Security of China issued the “Guiding Opinions on Conducting Pilot Programs for the Long-Term Care Insurance System,” which identified 15 cities as the first batch of LTCI pilot cities, while Jilin and Shandong provinces were designated as key liaison provinces. To further advance the development of the LTCI system and establish an independent insurance type, the National Healthcare Security Administration, together with the Ministry of Finance, issued the “Guiding Opinions on Expanding the Pilot Programs for the Long-Term Care Insurance System” in 2019. This expanded the number of pilot cities from the original 15 to a total of 49 cities, including the two key liaison provinces of Jilin and Shandong (Fig 1), aiming to create a social insurance system that funds long-term care for disabled individuals through mutual aid, covering basic daily living care and medical services.

**Fig 2:**
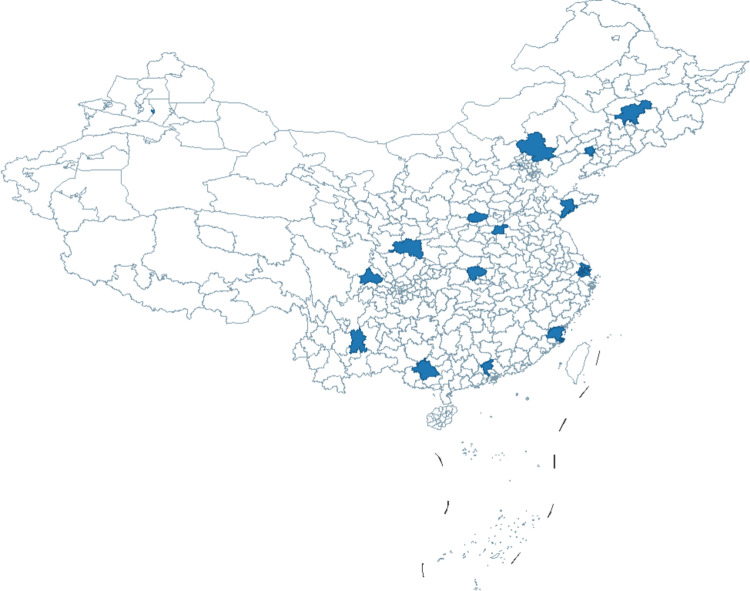
Sample cities.

Based on this, the selection of samples for this study follows China’s division of seven major geographical regions. A representative city from each region was selected for policy analysis: Northeast China, with Jilin Province as a key liaison province, selected Changchun as a representative of the first batch of pilot cities; in North China, Qingdao was chosen as it was the first to establish an LTCI pilot; East China selected Shanghai, a city with the dual status of the highest economic development level and a direct-controlled municipality; and Shijingshan District in Beijing, the only district-level pilot nationwide, was chosen as it holds significance for the broader rollout of the LTCI system. Additionally, to ensure a balance between the two batches of pilot cities and considering factors such as regional per capita GDP, aging rates, and the development of LTCI, 16 cities were selected for analysis, including Shihezi, Hanzhong, and Jingmen (Fig 2).

During the collection of long-term care insurance (LTCI) policies, the following retrieval strategies were employed to ensure the authority and comprehensiveness of the policy texts. First, policy documents were retrieved from the official websites of municipal governments, healthcare security departments, and human resources and social security departments in cities such as Changchun, Chengdu, Beijing, and Shanghai. Second, supplementary searches and verification of related policy documents were conducted through specialized databases such as “PKULaw” and the “China Long-Term Care Insurance Pilot Policy Database (CLIP).” After excluding notices, public announcements, news reports, and response letters, 66 valid policy documents were obtained. Given that the focus of this study is on the evaluation of LTCI policy design, 16 of the most recently issued special policy documents from each city were selected for analysis, as shown in Table 1.

**Table 1:** Summary of 16 long-term care insurance pilot policy texts.

No.	Policy Title	City	Document Number	Date of Issue
P1	“Implementation Opinions on Deepening the Long-Term Care Insurance System Pilot”	Chengdu	Cheng Fu Fa [2020] No. 16	May 2020
P2	“Jingmen Long-Term Care Insurance Measures (Trial)”	Jingzhou	Jing Zheng Fa [2016] No. 43	November 2016
P3	“Chengde Urban Employee Long-Term Care Insurance Management Measures”	Chengde	Cheng Yi Bao Zi [2021] No. 50	June 2021
P4	“Notice on Expanding the Pilot of the Medical Care Insurance System for the Disabled”	Changchun	Chang Yi Bao Fa [2021] No. 45	December 2021
P5	“Kaifeng Long-Term Care Insurance System Trial Measures”	Kaifeng	Bian Zheng [2020] No. 36	December 2020
P6	“Nanning Long-Term Care Insurance System Pilot Implementation Measures”	Nanning	Yi Bao Gui [2021] No. 1	January 2021
P7	“Kunming Municipal People’s Government Plan for Fully Launching the Long-Term Care Insurance System Pilot”	Kunming	Kun Zheng Fa [2020] No. 39	December 2020
P8	“Jincheng City Employee Long-Term Care Insurance Implementation Rules”	Jincheng	Jin Yi Bao Fa [2021] No. 1	February 2021
P9	“Qingdao Long-Term Care Insurance Measures”	Qingdao	Qing Yi Bao Fa [2021] No. 12	October 2021
P10	“Shanghai Long-Term Care Insurance Pilot Measures”	Shanghai	Hu Fu Ban Gui [2021] No. 15	December 2021
P11	“8th Division Shihezi City Long-Term Care Insurance Implementation Rules (Trial)”	Shihezi	Shi Shi Ban Fa [2017] No. 15	March 2017
P12	“Guangzhou Long-Term Care Insurance Trial Measures”	Guangzhou	Yi Bao Fa [2020] No. 37	December 2020
P13	“Hanzhong Long-Term Care Insurance Implementation Rules (Trial)”	Hanzhong	Han Zheng Ban Fa [2020] No. 25	November 2020
P14	“Fuzhou Long-Term Care Insurance Implementation Rules”	Fuzhou	Rong Yi Bao Wen [2021] No. 5	June 2021
P15	“Panjin City National Long-Term Care Insurance System Pilot Implementation Plan”	Panjin	Pan Zheng Ban Fa [2020] No. 25	December 2020
P16	“Shijingshan District Long-Term Care Insurance System Pilot Plan (Trial)”	Shijingshan	Shi Zheng Ban Fa [2018] No. 4	March 2018

**Fig 3:**
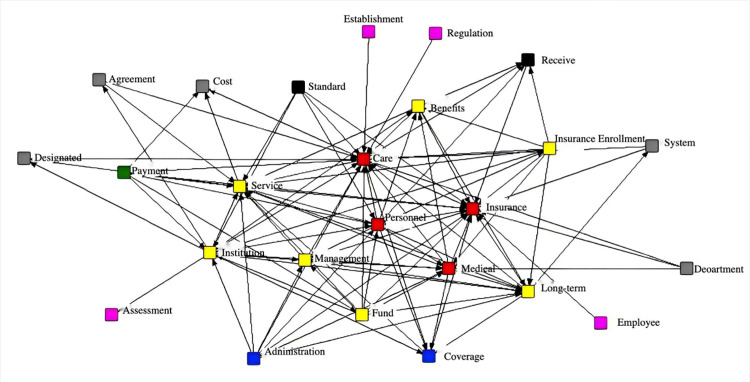
Social network analysis map of long-term care insurance policies.

Keyword Extraction and Social Network Analysis of Long-Term Care Insurance PoliciesUsing ROST CM software, keyword extraction and social network analysis were conducted on 16 long-term care insurance policy documents. The results are shown in Fig 3.

Social network analysis provides important references for the design of variables in the construction of the PMC index model. In the social network analysis map, high-frequency keywords from the long-term care insurance policies are organized into a node network, visually reflecting the structural relationships between these high-frequency terms. High-frequency words are represented as nodes, with the size of each node indicating its centrality strength. The more connections a node has with other nodes, the stronger its degree of centrality, indicating the greater importance of that node [49].

From Fig 3, it can be observed that: (1) “care,” “personnel,” “insurance,” and “medical” occupy central positions with the highest degree of centrality and the most connections to other keywords. These core themes indicate that the policy texts of long-term care insurance in various pilot cities focus on care, insurance content, medical services, and personnel. (2) Peripheral keywords include “services,” “funds,” and “enrollment,” which reflect the current focus of long-term care insurance policies on service content, fund operations, and the scope of enrollment. (3) Terms such as “payment,” “administration,” and “standards” indicate that long-term care insurance policies also address payment standards and the management of administrative agencies.

### 3.2 Variable settings

Following the modeling principles of the PMC index model, which emphasize multi-dimensional data processing through the use of multiple input-output matrices, this model requires consideration of the interaction between various factors and variables to assess the full impact of policies [50]. Additionally, the model is systematic and structured, with variables classified and parameters identified to emphasize the role and interrelations of each element, ensuring a clear and organized framework. The quantification and evaluation principle of the PMC index ensures that the model includes measurable standards for evaluating consistency and effectiveness, providing a scientific basis for decision-making. Finally, visualization and communication are integral, with the PMC surface (graphical representation) allowing for easier interpretation and understanding of model results, ensuring that policy recommendations are accessible to policymakers [50].

In line with these principles, variables X1 to X10 were established to construct the PMC index model for evaluating pilot policies of long-term care insurance. The secondary variables for the insured population (X1), covered groups (X2), payment standards (X4), involvement of commercial insurance (X5), financing channels (X7), financing methods (X8), and fund operation (X9) were defined based on the research by Wang [47]. The X3 variable was adjusted to include family caregiving services, which play a key role in long-term care insurance by offering flexible care options that reduce the burden on medical institutions and provide more personalized care in home settings. This adjustment is aligned with findings by Brendan,who emphasized that family health care is a critical component of long-term care insurance, addressing diverse needs such as daily living assistance, medical care, and psychological support for the elderly and disabled. However, it also faces challenges in terms of service quality, workforce training, and financial sustainability. The inclusion of this variable is based on the specific content of the pilot policy texts that highlight the growing importance of family caregiving services in providing cost-effective, quality care to individuals in home environments.

Additionally, service management (X6) and executive department (X10) variables were defined according to the social network analysis map of long-term care insurance policies. These variables reflect the core elements of long-term care insurance in China as outlined by Wang [47], who identified four key components that define the actual characteristics of LTCI: Capital (funding resources), Object (beneficiaries), Standard (quality evaluation criteria), and Supply (service provision). Regarding payment standards, the transparency and clarity of these standards are critical to ensuring the fairness and effectiveness of the LTCI system. As highlighted by Peng et al. [48], clear and transparent payment standards enable policymakers and the public to understand the coverage and conditions of insurance, which enhances the trust in the system and supports its long-term sustainability. The clarity of payment mechanisms is essential to avoid confusion and disputes, ensuring that beneficiaries can accurately assess their insurance entitlements and benefits. These components form the foundation for assessing the practical implementation and sustainability of LTCI policies.Ultimately, this includes 10 primary variables and 40 secondary variables, as detailed in Table 2.

**Table 2:** Variable settings.

No.	Primary Variable	No.	Secondary Variable	No.	Secondary Variable
X1	Coverage Scope	X1:1	Urban employees	X1:2	Aged 60 and above
		X1:3	Urban and rural residents	X1:4	Flexible employment
X2	Included Groups	X2:1	Severely disabled	X2:2	Moderately disabled
		X2:3	Severely cognitively impaired	X2:4	Moderately cognitively impaired
X3	Service Content	X3:1	Institutional care	X3:2	Home-based care
		X3:3	Hospital medical care	X3:4	Community day care
		X3:5	Family caregiving services		
X4	Payment Standards	X4:1	Clear payment amounts	X4:2	Clear payment proportions
X5	Commercial Insurance Participation	X5:1	Participates in management	X5:2	Provides evaluation and service products
X6	Service Management	X6:1	Standardized service institution management	X6:2	Strengthened staff training
		X6:3	Standardized insurance digitalization management	X6:4	Emphasis on third-party participation
		X6:5	Disability assessment and review		
X7	Financing Channels	X7:1	Transfer from medical insurance fund surplus	X7:2	Employer contributions
		X7:3	Individual contributions	X7:4	Government subsidies
		X7:5	Social donations	X7:6	One-time start-up funds
		X7:7	Allocation of welfare lottery funds		
X8	Financing Methods	X8:1	Fixed-amount financing	X8:2	Proportional financing
X9	Fund Operations	X9:1	Separate accounting management of funds	X9:2	Disability and cognitive impairment prevention fund
		X9:3	Long-term care insurance reserve fund/ risk contingency fund		
X10	Executive Departments	X10:1	Medical insurance department	X10:2	Civil affairs department
		X10:3	Finance department	X10:4	Human resources and social security department
		X10:5	Health department	X10:6	Tax department

### 3.3 PMC index calculation and analysis

Ruiz Estrada [50] outlined the specific steps for calculating the PMC index as follows:

Construct variables based on the policy texts, including primary and secondary variables, as shown in Equation (1):


X ～ N[0,1] (1)

Establish a multi-input-output table, and assign specific values to the secondary variables based on the binary method, as shown in Equation (2):


X= {XR:[0,1]} (2)

Calculate the values of the primary variables using the values assigned to the secondary variables in the previous step, as shown in Equation (3):


$${\text{X}}_{\text{t}}\left\{\overset{\text{n}}{\underset{\text{j}=1}{\sum }}\frac{{\text{X}}_{\text{tj}}}{\text{T}\left({\text{X}}_{\text{tj}}\right)}\right\},\text{t}=1,2,3,\ldots \ldots$$ (3)

In the equation, t represents the primary variable, and j represents the secondary variable.

Sum the values based on the above equations to calculate the PMC score for each policy, as shown in Equation (4).


$$PMC=\left[{\text{X}}_{1}\left(\overset{4}{\underset{\text{j}=1}{\sum }}\frac{{\text{X}}_{1\text{j}}}{4}\right)+{\text{X}}_{2}\left(\overset{4}{\underset{\text{j}=1}{\sum }}\frac{{\text{X}}_{1\text{j}}}{4}\right)+{\text{X}}_{3}\left(\overset{5}{\underset{\text{j}=1}{\sum }}\frac{{\text{X}}_{1\text{j}}}{5}\right)+{\text{X}}_{4}\left(\overset{2}{\underset{\text{j}=1}{\sum }}\frac{{\text{X}}_{1\text{j}}}{2}\right)+{\text{X}}_{5}\left(\overset{2}{\underset{\text{j}=1}{\sum }}\frac{{\text{X}}_{1\text{j}}}{2}\right)+{\text{X}}_{6}\left(\overset{5}{\underset{\text{j}=1}{\sum }}\frac{{\text{X}}_{1\text{j}}}{5}\right)+{\text{X}}_{7}\left(\overset{7}{\underset{\text{j}=1}{\sum }}\frac{{\text{X}}_{1\text{j}}}{7}\right)+{\text{X}}_{8}\left(\overset{2}{\underset{\text{j}=1}{\sum }}\frac{{\text{X}}_{1\text{j}}}{2}\right)+{\text{X}}_{9}\left(\overset{3}{\underset{\text{j}=1}{\sum }}\frac{{\text{X}}_{1\text{j}}}{3}\right)+{\text{X}}_{10}\left(\overset{6}{\underset{\text{j}=1}{\sum }}\frac{{\text{X}}_{1\text{j}}}{6}\right)\right]$$ (4)

The classification standards for policy evaluation results are set according to the method proposed by Estrada et al. (Table 3).

**Table 3:** PMC index evaluation standards.

PMC Index	[9, 10]	[7, 9)	[5, 7)	[1, 5)
Policy Evaluation	Perfect	Excellent	Acceptable	Poor

From this, the PMC index and policy grades for each digital economy policy are derived, as shown in Table 4.

**Table 4:** PMC index scores and policy grades for each policy.

No.	X1	X2	X3	X4	X5	X6	X7	X8	X9	X10	PMC Index	Grade
P1	0.5	0.5	0.6	0.5	1	0.8	0.71	1	0.33	0.83	6.77	Acceptable
P2	0.5	0.25	0.6	1	0.5	0.6	0.43	1	0.33	0.83	6.04	Acceptable
P3	0.5	0	0.6	1	0.5	0.8	0.29	0.5	0.33	0.67	5.19	Acceptable
P4	1	0.5	0.4	0.5	0.5	0.4	0.57	1	0	0.17	5.04	Acceptable
P5	0.75	0.25	0.8	1	1	0.8	0.57	1	0.33	1	7.50	Excellent
P6	0.5	0.25	0.4	1	0.5	0.4	0.14	0	0	1	4.19	Poor
P7	0.75	0.25	0.6	0.5	0.5	1	1	0.5	0.33	0.83	6.26	Acceptable
P8	0.5	0.25	0.6	1	0	0.8	0.86	0.5	0.33	0.83	5.67	Acceptable
P9	0.75	1	0.8	1	1	1	0.86	1	1.00	0.83	9.24	Perfect
P10	0.75	1	0.8	1	1	1	0.71	0.5	0.33	0.67	7.77	Excellent
P11	0.75	0.25	0.4	1	0.5	0.8	0.86	0.5	0.33	0.17	5.56	Acceptable
P12	1	0.5	0.6	1	0.5	1	0.71	0.5	0.33	0.83	6.97	Acceptable
P13	0.5	0.75	0.6	0.5	1	0.6	0.71	1	0.00	0.50	6.16	Acceptable
P14	0.5	0.25	0.6	0.5	1	0.8	0.57	0.5	0.33	0.83	5.89	Acceptable
P15	0.5	0.25	0.6	0.5	0	0.6	0.71	0.5	0.33	0.83	4.83	Poor
P16	0.5	0.25	0.6	1	0.5	0.8	0.57	1	0.33	0.83	6.39	Acceptable
Standard Deviation	0.18	0.28	0.12	0.24	0.33	0.19	0.21	0.30	0.22	0.24		

## 4. PMC index analysis of long-term care insurance

The PMC index of long-term care insurance policies from the 16 pilot cities indicates (Table 4) that one policy text achieved a “perfect” rating, two were rated as “excellent,” 11 were rated as “acceptable,” and two were rated as “poor.” The proportion of policies rated as “perfect” and “excellent” is 18.75%.

**Fig 4:**
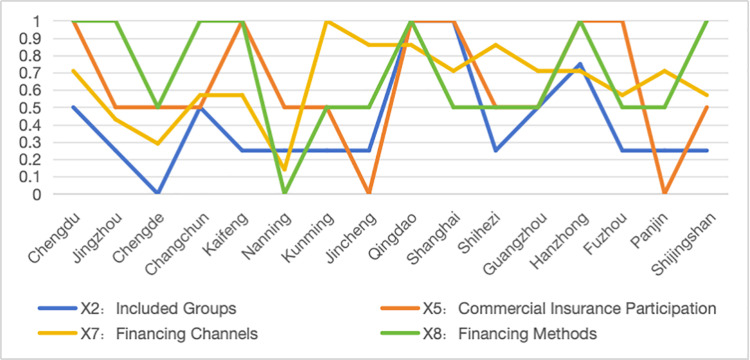
Line graph of PMC index distribution for the covered population (X2), commercial insurance participation (X5), financing channels (X7), and financing methods (X8) in pilot cities.

Regarding the local policies across various dimensions, Table 4 shows that the PMC values for commercial insurance participation (X5), financing methods (X8), and covered population (X2) exhibit the highest degree of variation, indicating significant differences in policy design across these dimensions. In terms of commercial insurance participation (X5), most cities allow commercial insurers to participate in the administration of long-term care insurance, but there is still no clear policy direction regarding the assessment of commercial insurance involvement and the provision of service products (Fig 4). This lack of clarity hinders the activation of private capital and the expansion of service diversity.

In May 2020, the National Healthcare Security Administration officially released the ‘Guiding Opinions on Expanding the Pilot Programs for the Long-Term Care Insurance System’ (Healthcare Security Administration Document [2020] No. 37) [National Healthcare Security Administration, Ministry of Finance, May 2020]. This document provided important guidance on the involvement of social forces in the administration of long-term care insurance and the role of commercial insurance, aiming to establish a multi-tiered long-term care security system that meets the diverse needs of the public. Specifically, it clarified that administrative management fees can be covered by the medical insurance fund, thereby creating opportunities for commercial insurance companies to participate in long-term care insurance. This encourages commercial insurance to play a role in addressing regional and demand differences, thus reducing the fiscal burden on the government.

The Guiding Opinions not only emphasize multi-tiered coverage and the integration of social forces into the long-term care system but also introduce a framework for shared responsibility in funding and service provision. These principles align with the PMC index model by ensuring that financial sustainability and system equity are at the heart of policy development. This document served as a critical foundation for the PMC index model by influencing the establishment of variables related to financing channels (X7), financing methods (X8), and fund operation (X9), which are crucial for evaluating the economic sustainability of LTCI systems.

Furthermore, the Guiding Opinions align with international best practices by promoting the integration of commercial insurance into the social insurance system, which is a concept also advocated in Germany’s long-term care insurance system. The PMC index model incorporates these insights, reflecting a hybrid financing model that combines public and private resources, ensuring the flexibility and scalability of LTCI systems to meet diverse population needs. By considering both local policy context and international experiences, the PMC index model is adapted to evaluate China’s LTCI pilot policies more effectively, taking into account both the institutional design and financial mechanisms that will support the system’s long-term sustainability.

Financing Channels (X7) and Financing Methods (X8) reflect the design of the financing mechanism.From the analysis of financing channels (X7), Jingmen, Chengde, and Nanning scored significantly lower (Fig 4). The vast majority of pilot regions rely on financial subsidies, contributions from employers and individuals, followed by the transfer of surplus from the medical insurance fund and social donations. However, consolidating a diversified financing mechanism does not mean accumulating as much capital as possible; instead, the focus should be on enhancing the efficiency of fund utilization, improving the protection of the insured, and maximizing the social benefits of the system. If expenditures are insufficient, it may increase the burden of fund management and operations.

In terms of financing methods (X8), the financing models for long-term care insurance can be divided into three types: proportional financing, fixed-amount financing, and mixed financing. Proportional financing refers to a system where different payers in the pilot regions contribute according to a set proportion based on a designated payment base. Pilot regions that have adopted proportional financing include Chengde, Kunming, Jincheng, Shanghai, Guangzhou, Fuzhou, and Panjin. Unlike proportional financing, fixed-amount financing stipulates that all payers must contribute a specific amount of premiums within a given period. Nanning and Shihezi are examples of pilot regions that use fixed-amount financing. Mixed financing combines both proportional and fixed-amount methods, tailored to different insured groups. Pilot regions using mixed financing include Chengdu, Jingmen, Changchun, Shanghai, Kaifeng, Qingdao, Hanzhong, and Beijing (Shijingshan District).

Fixed-amount financing does not vary according to personal income or economic status, making it simple to implement and resulting in lower administrative costs. Proportional financing, on the other hand, adjusts the contribution base according to economic growth and increases in personal income. Under equal benefits, higher-income individuals contribute more, thereby reflecting the social insurance function of income redistribution.

As shown in Table 4, the scores for service content (X3) and coverage (X1) are relatively concentrated across regions. In terms of service content, the services provided are fairly consistent among regions. However, it is worth noting that leading cities in the PMC index, such as Qingdao and Shanghai, lack a focus on family caregiving services in their policy texts. Family caregiving services refer to a model where relatives of the insured receive training and, upon certification, provide care to the insured, for which they are paid a monthly fee. By utilizing the care resources within the families of insured individuals and relieving them of the unpaid “caregiving role,” this approach extends the reach of services.

Regarding coverage (X1), the ratio of policies covering both employees and residents in the “perfect” and “excellent” categories is 1:1. For the other two secondary variables, the number of policies with assigned values is relatively low. Since the PMC index assumes equal weights for all primary variables, these differences did not affect the overall PMC index results.

A comparison of the long-term care insurance policy texts between the first and second batches of pilot cities reveals the following (as shown in Table 5): First, the scores for X2 and X7 are the same in both batches of cities, indicating that the expansion of the coverage groups was minimal and the financing channels were clearly defined. Second, the first batch of pilot cities scored higher on X1, X3, X4, X5, X6, X8, and X9 compared to the second batch. This is because the first batch of cities started their pilots earlier—Qingdao, for example, has had long-term care insurance policies for over 10 years. Additionally, the first batch includes two direct-controlled municipalities and two key liaison provinces, and Sichuan Province has also issued provincial-level guidance for the long-term care insurance pilots. As a result, the policy designs in the first batch pay more attention to detailed provisions regarding protection, administration, financing, and operations, which are comparatively more comprehensive than those in the second batch. In terms of coverage (X1), 87.5% of the first batch of pilot cities achieved universal coverage, while only 37.5% of the second batch included both urban and rural residents.

**Table 5:** Comparison of the average PMC index between the two batches of pilot cities.

Pilot Cities	X1	X2	X3	X4	X5	X6	X7	X8	X9	X10	Average PMC Index
First Batch	0.72	0.60	0.50	0.88	0.69	0.80	0.64	0.75	0.37	0.63	6.23
Second Batch	0.56	0.60	0.31	0.75	0.56	0.73	0.64	0.63	0.25	0.83	5.47

Third, the second batch of pilot cities scored higher on X10 (executive departments) than the first batch. The most notable difference is the involvement of tax departments in the second batch. In contrast, cities in the first batch, such as Shihezi and Changchun, only mentioned the healthcare departments in their policy texts, lacking coordinated issuance and integrated institutional arrangements. This fragmentation, with policies and funds spread across multiple sectors such as civil affairs, health, and disability organizations, often results in a lack of unified efforts, with each department acting independently. However, the “Guiding Opinions on Expanding the Pilot Programs for the Long-Term Care Insurance System” (Healthcare Security Administration Document [2020] No. 37) has brought about significant improvements in coordination among relevant departments and strengthened top-level design.

## 5. Analysis of representative policies based on the PMC surface

To more intuitively present policy scores, as well as their strengths and limitations, a PMC surface can be constructed based on the PMC index. The PMC index model for long-term care insurance policies contains 10 primary variables. Since most long-term care insurance policies cover urban employees, rural and urban residents, flexibly employed individuals, and people over the age of 60, variable X1 was excluded, and a 3 * 3 matrix was constructed as follows:


$$PMC=\left[\begin{array}{ccc} X_{1} & X_{4} & X_{7}\\ X_{2} & X_{5} & X_{8}\\ X_{3} & X_{6} & X_{9} \end{array}\right]$$

**Fig 5:**
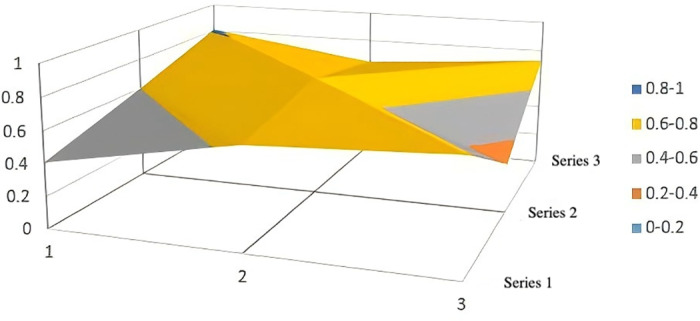
PMC surface of the virtual policy (P17).

**Fig 6:**
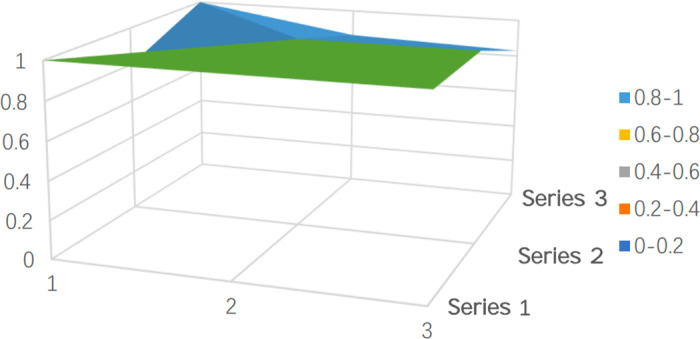
PMC surface of Qingdao (P9).

**Fig 7:**
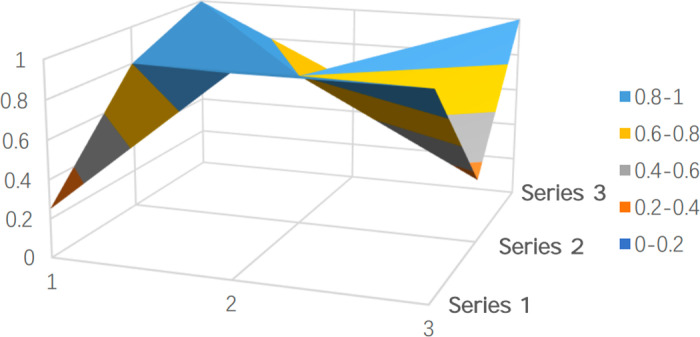
PMC surface of Kaifeng (P5).

**Fig 8:**
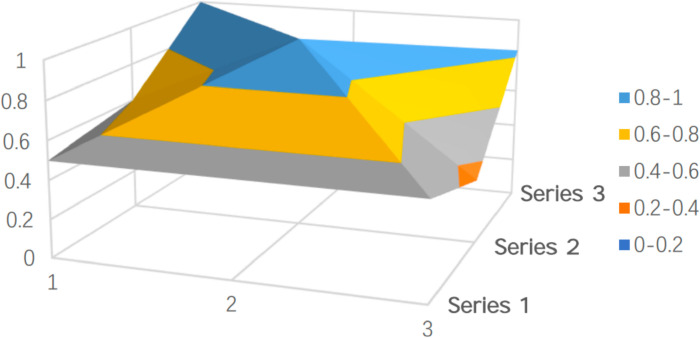
PMC surface of Guangzhou (P12).

**Fig 9:**
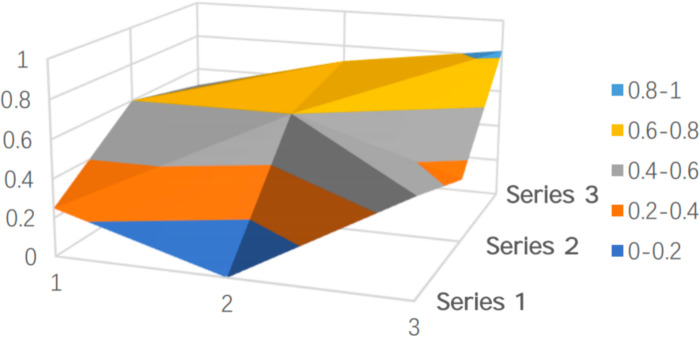
PMC surface of Panjin (P15).

To clearly and intuitively reflect the strengths and limitations of policy design in both batches of pilot cities and to showcase the results of the quantitative evaluation, representative policies were selected and their PMC surfaces were plotted. PMC surfaces are typically uneven 3D graphs, with different color blocks representing varying indicator scores. As shown in Figs 5–9, the raised areas in the surface graph indicate higher scores for the primary variables, while the recessed areas indicate lower scores.

The PMC index model selected 10 primary variables and 40 secondary variables, and the values of the primary variables were calculated using the PMC index model formulas. By calculating the standard deviations and variances of these primary variables, the PMC values for 16 cities were obtained. Four cities were selected as representative policies, based on the PMC index criteria and the actual situations of the pilot cities. The representative policies include: (1) Qingdao (P9), from the “perfect” category, (2) Kaifeng (P5), from the “excellent” category, (3) Guangzhou (P12), from the “acceptable” category, and (4) Panjin (P15), from the “poor” category.

Additionally, to provide an overall impression of the 16 sample cities and facilitate better comparisons, a virtual policy (P17) was constructed based on the average values of the primary variables from the 16 selected policies in the sample cities, as shown in Fig 5.

The average PMC index value calculated for the 16 prefecture-level cities is 6.217, which falls within the “acceptable” range and approaches the “good” category. From the shape of Fig 5, despite the overall average evaluation being “good,” the surface is relatively smooth, indicating that, on average, the internal consistency of long-term care insurance policies across the 16 pilot cities is high, with balanced development across various aspects of the policy, leading to a reasonably structured system.

Compared to Fig 6, the values for Qingdao’s 9 primary variables are significantly higher than the average of the 16 cities. Six variables—covered population (X2), payment standards (X4), commercial insurance participation (X5), service management (X6), financing channels (X7), and fund operations (X8)—all scored full marks. Especially for the covered population (X2) variable, which scored full marks, analysis of Qingdao’s policy shows that although the 2012 version of Qingdao’s long-term care insurance (1.0) did not explicitly define the categories of disabled and cognitively impaired elderly, it proposed two relatively vague concepts: (1) Insured individuals who, due to old age, illness, or disability, have lost some or all bodily functions, are bedridden for long periods, and are unable to care for themselves, requiring long-term medical care in designated care institutions; (2) Insured individuals who, due to old age, illness, or disability, have lost some or all bodily functions, are bedridden for long periods, and require medical care to be provided at home due to changes in their condition. These concepts have gradually evolved over time, with individualized service requirements emerging for disabled and cognitively impaired elderly. In response, Qingdao’s 2018 version (2.0) of the long-term care insurance policy made detailed distinctions between these two groups to ensure more precise services.

Compared to other sample cities, Qingdao’s financing channels (X7) reflect a diversified funding model. Not only does it rely on the medical insurance fund pool, but it also balances payment standards (X4) and financing methods (X8), reflecting a well-coordinated approach to fund operations. This allows Qingdao to provide higher quality and more refined services, indirectly improving the quality of long-term care services (X3) in the region. Furthermore, Qingdao has established detailed regulations on fund evaluation and supervision, which have contributed to a perfect score in the fund operations (X9) variable. Among all the sample cities, Qingdao is the only one with a perfect score in this variable. In addition to maintaining separate accounts and audits, Qingdao has also made detailed plans for funding the prevention of disability and cognitive decline. The “Notice on Issuing the Qingdao Long-Term Care Insurance Plan” in 2021 [Qingdao Municipal Government Office, March 2021, “Notice on Issuing the Qingdao Long-Term Care Insurance Plan” stipulates, “A prevention and delay fund for disability and cognitive decline shall be established, with up to 3% of the annual long-term care insurance funds for employees and residents allocated for this purpose.” This demonstrates that Qingdao not only excels in differentiating services for disabled and cognitively impaired elderly, but also places great emphasis on preventive measures for these conditions.However, it should be noted that although Qingdao has differentiated payment ratios for different contribution levels, it has not yet implemented a “longer contribution, higher benefits” system, which may represent a limitation in the policy.

Based on the above analysis, it is evident that Qingdao’s long-term care insurance policy is relatively well-developed and reasonable, with a well-established service mechanism. According to available policy documents and other materials, Qingdao, as one of the key pilot cities in the first batch of long-term care insurance trials, launched its long-term medical care insurance system (referred to as “long-term medical care system”) in 2012, ahead of the rest of the country. Prior to this, in response to the central government’s call to streamline administration and delegate power, Qingdao established community-based medical insurance fund management offices in 2005 and began developing community healthcare institutions and home care beds. In 2006, elderly medical care was incorporated into the community medical insurance system, and nursing homes and elderly care institutions with qualified medical care services were included in the medical insurance network. With the increasing pressure of health service demand and medical care resources brought by the aging population, Qingdao’s approach—aimed at both improving the efficiency of medical resources and funds, and providing more targeted and adaptable basic medical care services—was formulated after multiple rounds of calculation and coordination with various departments. Ultimately, the city proposed the establishment of a long-term medical care insurance system within the framework of the urban basic medical insurance system, starting a pilot program in July 2012. Qingdao’s policy has evolved over nearly a decade, continuously improving and adapting based on practical implementation, with a goal of achieving diversified, personalized, and humanized services. This analysis shows that Qingdao provides a relatively complete and practical blueprint for long-term care insurance system design, offering valuable lessons for other cities.

Kaifeng ranks second among the policies classified as ‘excellent,’ just behind the economically developed city of Shanghai, with a PMC index value of 7.50, nearly one point higher than the average, placing it in the ‘excellent’ category (Fig 7). As a second-batch pilot city, Kaifeng officially introduced its long-term care insurance policy in 2021. According to the Kaifeng municipal government’s website, relevant departments involved in the formulation of Kaifeng’s long-term care insurance policy conducted multiple field visits to first-batch cities such as Shanghai and Nantong in 2020 to learn from their advanced experiences. Based on this research, Kaifeng issued the ‘Trial Measures for the Long-Term Care Insurance System of Kaifeng’ on December 29, 2020.

Compared to Fig 5, the policy design in Kaifeng is relatively reasonable. Except for the covered population (X2) and financing channels (X7), which are below the average, the other seven primary variables are above the average, with four primary variables scoring full marks. The average score for the covered population (X2) is only 0.25. Among the 16 sample cities, only Qingdao, Hanzhong, and Shanghai include the cognitively impaired population, while the other 13 pilot cities only address the physically disabled, which may lead to a lack of targeted services and measures in the future, affecting the precision and quality of long-term care services.

In terms of financing channels (X7), Kaifeng has not established clear regulations for converting the medical insurance fund into the long-term care insurance fund, which could increase the financial burden on individuals participating in long-term care insurance and hinder the long-term sustainability of the policy. However, on the other hand, not relying heavily on medical insurance funds reduces the risks associated with the financing mechanism. Meanwhile, Kaifeng scored full marks for the financing methods (X8), as it balances both fixed-amount and proportional financing, addressing both fairness and efficiency, and facilitating easier management and calculations.

Regarding service content (X3), Kaifeng’s policy specifies, ‘Home-based autonomous care refers to the form of care where a family member of the insured, after receiving qualified training, provides care for the insured. The maximum monthly payment from the long-term care insurance fund is 900 yuan per person’ [Kaifeng Municipal Government Office, December 2020, ‘Trial Measures for the Long-Term Care Insurance System of Kaifeng’]. This inclusion of family caregiving services innovates the service content and also specifies that ‘during the period of receiving benefits, the insured may choose one form of care service, and the service type can be changed,’ which further reflects the humanization and flexibility of the policy.

It is undeniable that Kaifeng’s high PMC index score, surpassing that of more developed regions, is due to strong government support. The Kaifeng municipal government has played a pivotal role in ensuring the successful implementation of the long-term care insurance system through effective policy framework and system building. The local government introduced a comprehensive set of policies and regulations, including the issuance of 19 policy documents, such as the Trial Measures for the Long-Term Care Insurance System of Kaifeng. Additionally, Kaifeng’s financing model, incorporating multiple funding sources like unit and individual contributions and fiscal subsidies, has contributed to the program’s financial sustainability.

Furthermore, Kaifeng has taken significant steps to raise public awareness and promote participation in the program. According to the Kaifeng Medical Security Bureau’s survey, 9388 residents benefiting from the long-term care insurance expressed satisfaction with the policy, citing improvements in their quality of life and reduced financial burden. As of September 2023, 420,000 people were enrolled, and 18,307 people were receiving benefits, with a total payout amounting to 145 million yuan. The program has not only reduced the financial burden on families of disabled individuals but also significantly improved their living standards, which has led to increased public approval and positive feedback. The introduction of a positive policy feedback mechanism has further enhanced resident satisfaction, demonstrating the government’s commitment to providing sustainable and effective care for its citizens.

Compared to Fig 5, Guangzhou scores (Fig 8) below the average on two primary variables: commercial insurance participation (X5) and financing methods (X8), while its service content (X3) matches the average. The other six primary variables score above the average. Guangzhou’s PMC index value is 6.97, slightly above the average. As one of the first-batch pilot cities, Guangzhou officially launched its long-term care insurance program in 2017 and has gone through three stages: initiation, development, and deepening. In 2021, Guangzhou fully reformed its long-term care insurance system based on the Guiding Opinions on Expanding the Pilot Programs for the Long-Term Care Insurance System.Regarding commercial insurance participation (X5), Guangzhou’s policy lacks detailed provisions. The policy only mentions commercial insurers’ involvement in administration but does not provide specific guidelines for operations or service provision. There is limited regulation on the role of commercial insurance in providing services, assessing disability, and managing funds. This insufficient involvement of commercial insurance results in a single source of funding, inadequate risk-sharing, and a lack of service diversity. The core issue lies in the fact that Guangzhou’s policy remains heavily government-driven, with a tendency toward risk control. As a result, it relies more on the medical insurance fund and fiscal support, while avoiding the potential uncertainties of involving commercial insurers, such as management issues, increased regulatory costs, or conflicts of interest.

In terms of financing methods (X8), Guangzhou adopts proportional financing, which ensures fairness but poses challenges in regulation. There is a gap between income and expenditure, and the lack of a hybrid financing model (combining fixed-amount and proportional financing) reduces the policy’s flexibility and makes it difficult to cater to insured individuals with different economic backgrounds. Guangzhou’s financing mechanism heavily depends on the transfer of surplus from the medical insurance fund, neglecting fiscal appropriations and social donations. This increases the burden on the medical insurance fund, with no flexible financing mechanism in place. Moreover, the policy does not set differentiated financing standards for groups with different income levels, leading to lower participation rates among low-income groups.

Overall, the weaknesses in commercial insurance participation and financing methods result in Guangzhou’s long-term care insurance policy scoring lower than excellent examples like Qingdao. The main issues in Guangzhou’s policy are the lack of market involvement and a single financing mechanism. Guangzhou should learn from the perfect and excellent pilot cities by focusing on improving policy stability, enhancing disability assessment accuracy, and encouraging commercial insurers to develop long-term care products. Future improvements should target expanding commercial insurance participation, introducing a hybrid financing model, and strengthening support for family caregiving services. Specific measures could include enhancing administration, diversifying care services, and expanding product offerings.

Fig 9 shows that Panjin’s PMC index ranks at the lower end of the sample, placing it in the “poor” policy category. The surface chart reveals significant depressions, with only two primary variables—payment standards (X4) and executive departments (X10)—scoring above the average, while the rest are below average. Particularly for commercial insurance participation (X5), Panjin scores 0, indicating that its policy did not consider the importance of commercial insurance participation or, due to local circumstances, was unable to implement it. The lack of involvement from commercial insurance and other third parties may affect service quality and the orderly operation and improvement of the long-term care insurance system. Panjin has a weaker economic foundation, and the smaller scale of its economy limits its ability to attract commercial insurers. Additionally, local governments may be concerned that commercial insurance participation could lead to higher premiums and unstable service quality. Combined with conservative policy design, Panjin’s long-term care insurance remains government-dominated, with minimal risk-taking.

Financing channels (X7) also show certain limitations. Panjin’s policy relies heavily on the surplus of the medical insurance fund and does not fully utilize diversified financing channels such as fiscal appropriations, social donations, and charitable funds. As a small to medium-sized city, Panjin faces significant pressure on its medical insurance fund, and over-reliance on surplus funds could lead to financial strain, affecting the normal operation of the medical insurance system. Additionally, local fiscal resources are limited, and the lack of sufficient local fiscal investment makes it difficult to support long-term care insurance on a larger scale. Panjin’s financing structure limits the sustainability of its policy and leads to financial stress. This issue aligns with a common challenge in China’s long-term care insurance pilots—single-source financing that fails to introduce more market forces or social donations to alleviate financial pressure.

For small to medium-sized cities, two key issues are insufficient fiscal support and limited financial resources, as well as the lack of family caregiving support and community care models. The service content remains relatively singular, unable to meet diverse needs. This also leads to lower scores in covered population (X2), as the policy does not clearly differentiate among the covered groups. This is similar to the difficulties faced by many small to medium-sized pilot cities nationwide, where most resources are concentrated on institutional care, while more cost-effective home-based care models are overlooked. The underlying reason is that smaller local governments lack sufficient resources to develop diverse care services, particularly family caregiving. Additionally, their limited experience in promoting home-based and community care has resulted in incomplete policies and insufficient execution capabilities.

The low PMC index scores in poor-performing cities are partly due to the late implementation of long-term care insurance and limited development experience, and partly due to lower levels of economic development.

From the analysis of the four representative policies, we can conclude that Qingdao, which falls under the “perfect” category, scores near full marks for all variables except for the absence of specific provisions on family caregiving services. Kaifeng, in the “excellent” category, has shortcomings in covered population, as it does not include the cognitively impaired elderly, and its disability assessment is not sufficiently detailed. Additionally, its financing channels are limited, and it has not fully utilized the medical insurance fund during its initial stages of operation. Guangzhou, in the “acceptable” category, lacks detailed provisions on commercial insurance participation, which could lead to unclear responsibilities and gaps in service content. Finally, Panjin, in the “poor” category, faces numerous challenges, with the most significant being the lack of commercial insurance participation in the operation of long-term care insurance, which needs continuous improvement.

Fundamentally, the differences in policy content between pilot cities reflect variations in local governments’ understanding of long-term care insurance and their capacity to provide protection. The design of institutional plans also reflects the practical direction of local decision-makers. Cities with lower PMC index scores should tailor policy improvements based on their specific development context and indicator performance, in order to better guide the practice of long-term care services.

## 6. Conclusions and suggestions

In reviewing the entire process of establishing the long-term care insurance policy evaluation index system and PMC surface using the PMC index model, the analysis was divided into two parts: an analysis of the ten policy evaluation dimensions and an analysis of the degree of improvement in representative policies from both batches of pilot cities. The study also ranked the policies of the 16 pilot cities comprehensively. Finally, it identified the essential problems in specific indicators of long-term care insurance policies and highlighted areas for broader implementation. The practice of long-term care services requires policy support and protection, and the PMC index clearly reveals the practical functioning of the long-term care insurance system. Based on the model construction, five recommendations were identified as having a positive effect on improving the long-term care insurance system.

### 6.1 Improve caregiver support policies and enhance the quality of family caregiving services

Although the analysis results of the PMC index model showed that Shanghai did not meet the family caregiving service policy standards, as early as 2005, Shanghai proposed the “9073” elderly care service model. This model emphasizes that 90% of elderly care is provided by the family, 7% through community home care services, and 3% through institutional care services. This approach takes into account both the adaptability of the elderly to their environment and the issue of economic burden, and it remains a widely advocated model in long-term care insurance systems. However, the PMC index model revealed several issues with family caregiving services within the broader range of care services, including institutional care, home-based care, hospital medical care, community day care, and family caregiving services.

From the analysis of the relationship between commercial insurance participation and family caregiving services across the 16 pilot cities, it was found that most cities have linked family caregiving services to commercial insurance participation [51–53]. Since commercial long-term care insurance is fully voluntary, where both the insured and the financing entity are individuals, the role of family decision-making becomes particularly important.

Among the five cities that comply with family caregiving service policies—Chengdu, Kaifeng, Kunming, Jincheng, and Beijing—each requires professional caregiver training as a prerequisite. In this regard, developed countries such as Germany, Japan, and the United States have promoted family caregiving through different systems: broad mandatory public long-term care insurance, mandatory public long-term care insurance for specific groups, and voluntary commercial long-term care insurance. Scholars have compared these systems and highlighted their distinctions (see Table 6). Among the three, the U.S. long-term care insurance system was found to be the most effective, followed by Germany, with Japan ranking third [54].

**Table 6:** Differences between public LTCL and commercial LTCL.

Comparison Category	Public Long-Term Care Insurance (LTCL)	Commercial Long-Term Care Insurance (LTCL)
Nature of Insurance	State-guaranteed, non-profit	Commercial, profit-driven
Insured Population	Entire population	Individuals who purchase insurance
Insurance Participation	Mandatory	Completely voluntary
Financing of Insurance	Joint contributions from employees and employers	Individual policyholders
Responsible Entity	Government	Insurance companies
Level of Protection	Focus on “protection”	Focus on “reimbursement”

Based on these insights, this study draws on the experience of the U.S. voluntary commercial long-term care insurance system, supplemented by Germany and Japan’s mandatory public long-term care insurance models. It emphasizes the role of national policy in guiding long-term care insurance and suggests that China could benefit from integrating more flexible commercial insurance options to better support family caregiving services. Additionally, it is crucial to coordinate the flexibility of commercial insurance with the mutual assistance of public insurance, establish a comprehensive third-party review agency, implement strict disability assessment standards, and actively leverage market mechanisms.

### 6.2 Expand funding channels and enhance the fairness of financing mechanisms

The results of the PMC index model indicate that the policies of Nanning, Chengde, and Jingmen perform poorly in terms of financing channels, with structural imbalances between conventional and unconventional financing methods. Additionally, Nanning’s overall policy is weak in terms of financing methods, where internal structural inconsistencies are evident.

A review of long-term care insurance financing mechanisms abroad reveals that Germany, Japan, and South Korea primarily adopt social insurance, commercial insurance, allowance-based, or mixed models, forming stable long-term care insurance financing systems involving individuals, employers, and the government [11]. In contrast, China’s long-term care insurance financing mechanism has evolved from the cooperative medical system of the 1950s to the urban and rural residents’ basic medical insurance, a system that merged the new rural cooperative medical system and the urban residents’ basic medical insurance [55]. This merged system retained the basic model of the earlier medical financing systems, where each person pays a fixed amount of medical insurance. According to statistics from the National Healthcare Security Administration, from 2016 to 2021, individual contributions to China’s urban and rural residents’ medical insurance increased from 150 yuan per person per year to 320 yuan per person per year, with an average annual increase of about 30 yuan. Although this fixed-amount financing method is equal in contributions and easy to implement, it essentially mirrors the financing model of commercial insurance in a market-based system [11]. Commercial insurance is a market transaction between the insurer and the insured, governed by market economic principles. It does not account for the varying payment capacities of policyholders and charges fixed premiums based on individual risk and insurance needs [13].

China’s urban and rural residents’ basic medical insurance is a government-led social basic medical insurance system targeting all non-employed residents, and adopting the commercial insurance financing model would be inappropriate. Fixed-amount financing results in unequal payment burdens for different income groups, creating a reverse adjustment effect where low-income groups “subsidize” higher-income groups, violating the principle of fairness in “ability-to-pay” financing.

The core value of modern social security is fairness, and promoting social equity is a fundamental goal of social security. As an essential aspect of social security, social insurance should ensure fairness at the starting point, maintain fairness in the process, and strive to reduce inequalities in outcomes among social members. Social insurance funds not only provide economic protection for society’s members but also help regulate income distribution. Thus, social insurance should be financed based on individuals’ capacities, with higher-income earners paying more and lower-income earners paying less. The principle of “contributing based on ability and receiving based on need” can effectively adjust income distribution, reduce income disparities between different groups, and promote social equity.

In principle, proportional financing should be calculated based on household disposable income per capita, with a specific percentage of that income set for contributions. Furthermore, support should be provided to low-income families, such as lowering their contribution rates or increasing fiscal subsidies, to promote equity in financing and fairness in the system.

### 6.3 Rationally allocate insurance policy resources and gradually eliminate policy barriers

The PMC index analysis of the coverage and executive departments of long-term care insurance reveals several issues, including uneven distribution of resources between urban and rural elderly, resource waste, and discrepancies in medical insurance benefits between different parts of the same city. Specifically, among the 16 pilot cities examined in this study, only Changchun and Guangzhou had relatively balanced policy resource allocation, while the others exhibited varying degrees of imbalance [56,57].

For urban employees, the policies of all 16 pilot cities addressed the issue, clearly specifying that those covered by social medical insurance should also participate in either employee or resident long-term care insurance. Comparing Germany’s coverage and protection of insured individuals, the German long-term care insurance system achieves universal coverage (Table 7) [57], and the range of beneficiaries is gradually expanding, with both the number and proportion of beneficiaries increasing. In contrast, China’s long-term care insurance covers a smaller population (see Table 8), with the main beneficiaries being severely disabled elderly individuals. Most regions do not extend coverage to moderately or mildly disabled individuals, nor to those with cognitive impairments.

**Table 7:** Full coverage of Germany’s LTCI system.

Classification of Medical Insurance Participants	Long-Term Care Insurance Coverage Status
Members of Sickness Fund Associations	Automatically enrolled in social long-term care insurance; spouses and children are automatically covered by the system
Participants in Private Health Insurance (annual post-tax income above a certain threshold requires purchase)	May choose to purchase private long-term care insurance or voluntarily enroll in social long-term care insurance
Other individuals who voluntarily join the social health insurance system	Automatically enrolled in social long-term care insurance

**Table 8:** Comparison of financing entities in China’s LTCI pilot regions.

Financing Entity	Pilot Regions
Urban Employee Medical Insurance	Qiqihar, Anqing, Shangrao, Jingmen City, Ningbo, Chengde, Guangzhou, Chongqing, Chengdu
Urban Employee & Urban Resident Medical Insurance	Changchun, Qingdao, Nantong, Shihezi
Urban Employee & Urban-Rural Resident Medical Insurance	Shanghai, Suzhou, Shijingshan District Beijing

Currently, as the long-term care insurance system is still in the pilot stage in the 16 regions, the government can address the issue of geographical inequity in care resources by coordinating the overall layout and providing policy incentives for the strategic placement of long-term care service institutions. Based on the care service needs of insured individuals in different areas, the government can integrate internal care resources, promote the integration of care institutions within each region, and encourage collaboration and communication between institutions. This would facilitate resource sharing and information exchange. At the same time, insufficient overall resources remain a major constraint on the utilization of long-term care services, and increasing resource investment is the primary way to resolve this issue. Thus, it is crucial to encourage qualified institutions to participate, reasonably set entry standards, and provide policy incentives such as tax reductions to alleviate financial burdens.

### 6.4 Increase attention to the cognitively impaired elderly and clarify the standards for determining “disability” in elderly individuals

An analysis of the PMC index for the covered population indicates that many policy documents focus on elderly individuals with disabilities, particularly those with severe disabilities, while attention to the cognitively impaired elderly remains limited, regardless of the severity of their condition. Furthermore, while policies involving medical insurance departments, civil affairs departments, and finance departments are relatively well-developed, those from human resources and social security departments, as well as health departments, require further improvement. Although current long-term care insurance policies emphasize elderly individuals with disabilities, there is an urgent need to increase focus on cognitively impaired individuals and develop corresponding measures.

At the same time, the potential for the reversal of “disability” in elderly individuals and the flexible transition between severe and mild disability highlight the lack of a reasonable standard for determining “disability” in current policies. This leads to procedural issues in policy formulation. Additionally, the relatively low attention given to cognitively impaired individuals further complicates the practical aspects of developing long-term care insurance policies for this group.

Germany’s care levels are divided into three categories: moderate, severe, and very severe care needs [7]. In comparison, China’s long-term care insurance primarily covers only those with severe disabilities. In Germany, after submitting a claim, the insured is assessed through an in-home care evaluation conducted by the medical insurance review agency, which determines the appropriate care level based on the results. Germany’s approach can help address the issue of defining disability in China, especially given the flexibility in how disability is assessed. Germany’s long-term care insurance also follows the principle that home care takes precedence over institutional care (Table 9), with home care receiving more policy support than institutional care.

**Table 9:** Regulations on long-term care insurance benefits in Germany.

Care Level	Home Care		Full Institutional Care
	In-Kind Benefits from Care Institutions	Care Allowance when Arranging Own Care	
Ⅰ	420	215	1023
Ⅱ	980	420	1279
Ⅲ	1470	675	1470
Ⅳ	1918		1759

Unit: Euros

Therefore, based on the German experience, China’s long-term care insurance system should consider expanding benefit eligibility to include individuals with moderate disabilities, aiming to achieve broad coverage. At the same time, regarding the issue of disability standards, China could draw lessons from Germany’s experience. For cognitively impaired individuals, Germany’s principle of prioritizing home care over institutional care could offer valuable insights [7].

### 6.5 Strengthen the preventive role of long-term care insurance and progressively expand its coverage

In terms of the service content of long-term care insurance, from family caregiving services, institutional care, hospital medical care, to community day care and finally home-based care, the scope of services and delivery methods are continually expanding. Regarding service management, the system plays a preventive role by standardizing the management of service institutions, strengthening the training of caregivers, promoting the digitalization of insurance management, encouraging third-party participation, and implementing re-evaluation of disability assessments. These measures influence the various stakeholders—both service providers and beneficiaries—within different fields. Regarding fund operations, dedicated accounts, the establishment of a disability and cognitive impairment prevention fund, and a long-term care insurance reserve fund (risk contingency fund) continue to expand their inherent functions.

For the pilot cities, long-term care insurance is still in the experimental phase, where the approach has been described as “crossing the river by feeling the stones.” In Qingdao, the first pilot city, the system initially covered only medical care. However, as the pilot reforms deepened, coverage expanded to include both medical care and daily living assistance. Medical care primarily focuses on health management and maintenance treatments, while daily living assistance includes long-term care, rehabilitation training, palliative care, end-of-life care, and emotional support, covering a total of 61 different services.

From the experiences of Japan and Germany, which have established sustainable long-term care insurance systems, both countries have developed relatively mature operational models in terms of financing mechanisms and service provision [11]. Specifically, Japan’s long-term care insurance is funded by a combination of government taxes, long-term care insurance contributions, and individual payments. From the supply-side perspective, Japan has divided care services into health promotion, care prevention, rehabilitation, home care, and institutional care. In Germany, social long-term care insurance is financed through a pay-as-you-go system, with the principle of home care taking precedence over institutional care in terms of service provision. These countries have thus reached a relatively advanced stage in both financing and service provision.

The rich experiences of Japan and Germany in terms of financing mechanisms and service provision can serve as excellent models for establishing and improving China’s long-term care insurance system [10]. However, it should also be noted that both countries face issues such as insufficient insurance reserves, a shortage of caregivers, and complex administrative processes in their long-term care insurance systems. Therefore, while China can learn from international experience, it must also adapt these lessons to its national context and continue to adopt a “crossing the river by feeling the stones” approach.

Given the deepening aging of China’s population, the rate of funding growth for commercial long-term care insurance may slow. At the same time, upon reflection, it becomes clear that there are numerous issues with family caregiving services. In this context, commercial insurance participation can play a positive role by helping manage operations and providing products and evaluations [7]. This can effectively alleviate the current pressures on public long-term care insurance and further expand the scope of coverage.
